# Multiparameter ranking of carbazoles for anti-trypanosome lead discovery

**DOI:** 10.3389/fddsv.2024.1430927

**Published:** 2024-08-14

**Authors:** Amrita Sharma, Carlos E. Sanz-Rodriguez, Michael P. Pollastri, Andrei Purmal, Kojo Mensa-Wilmot

**Affiliations:** 1 Department of Molecular and Cellular Biology, Kennesaw State University, Kennesaw, GA, United States; 2 Center for Tropical and Emerging Global Diseases, University of Georgia, Athens, GA, United States; 3 Department of Chemistry and Chemical Biology, Northeastern University, Boston, MA, United States; 4 Incuron, Inc., Buffalo, NY, United States

**Keywords:** trypanosomiasis, mouse model of disease, hit-to-lead selection, drug modes of action, parasite, infectious disease

## Abstract

The criteria for the progression of hits in the discovery of leads for human African trypanosomiasis (HAT), a neglected disease caused by the microbial eukaryote *Trypanosoma brucei*, are not standardized. Hits are advanced upon meeting thresholds for drug-like molecules. Following those principles, pharmacokinetics (C_max_ and AUC_0–6h_) and anti-trypanosome characteristics predicted the arrest of *T. brucei* proliferation in mice by three curaxins. Unexpectedly, while CBL0137 cured HAT in a mouse model, CBL0174 and CBL0187—structural analogs of CBL0137 with similar drug-like properties—failed to control *T. brucei* division. We here propose an alternative strategy that integrates physicochemical, metabolic, pharmacokinetic, pharmacodynamic, tissue distribution, and trypanocidality parameters into calculating a score for ranking compounds in hit-to-lead campaigns. Data from our studies of curaxins support the feasibility of this goal. Serum dropped the anti-trypanosome potency of CBL0174 and CBL0187 considerably. Delayed trypanocidal concentrations (DTC_25_ and DTC_90_) were used to study modes of curaxin actions in trypanosomes. Efficacy of CBL0137 in mice correlated with (i) a high AUC_0–6h_: DTC_90_ ratio, (ii) blocking of transferrin endocytosis, and (iii) the inhibition of protein synthesis. Hydroxylation of the carbazole prevented CBL0137 from inhibiting endocytosis of transferrin. The multiparametric score “Curaxin HAT lead efficacy (CHLE)” score was calculated using pharmacokinetic, physicochemical, metabolic, brain exposure, and pharmacodynamic data; CBL0137 was the highest scoring hit. Complementing these observations and predictive of performance of curaxins in mice, CBL0137, but not CBL0174 or CBL0187, was trypanocidal after the exposure of trypanosomes to AUC_0–6h_ amounts of the hits for 6 hours *in vitro*. We discuss a role for CHLE scores in ranking curaxins for anti-HAT lead discovery. The principles used to develop CHLE scores may be used to calculate new ones for other scaffolds during the discovery of leads for HAT or other infectious diseases.

## Introduction

Human African trypanosomiasis (HAT), a threat to ~60 million people in the countries of Sub-Saharan Africa, is caused by the protist *Trypanosoma brucei* sp. (Hasker et al., 2022). The trypanosome life cycle involves transit through a tsetse fly vector in which bloodstream trypanosomes differentiate into an insect-stage form (procyclic) by expressing a different surface coat protein (Bangs, 2018; Faria et al., 2022) and altering several metabolic pathways (Michels et al., 2021). Under natural conditions, *T. brucei* is found in many host tissues, including the brain (Crilly and Mugnier, 2021). Switching of the major coat protein (variant surface glycoprotein) during “antigenic variation” (Bangs, 2018; Faria et al., 2022) poses a significant impediment to vaccine development. Consequently, HAT is managed with chemotherapy.

Of the limited number of drugs for treating chronic HAT, melarsoprol is toxic and others require frequent intravenous administration (Dickie et al., 2020). Fexinidazole is the first oral drug developed for chronic HAT in 40 years (Deeks, 2019; Pelfrene et al., 2019). Reports of trypanosome resistance to fexinidazole or nifurtimox (Sokolova et al., 2010; Wyllie et al., 2016; Kasozi et al., 2022) draw attention to a persistent threat posed by parasite “accommodation” of drug pressure, highlighting a need to sustain development of new chemical entities to combat HAT (Baker and Welburn, 2018; De Rycker et al., 2018).

The carbazole derivative CBL0137 (1-[6-acetyl-9-[2-(isopropylamino)ethyl]carbazol-3-yl]ethanone) (Burkhart et al., 2014) cured HAT in a mouse model of acute disease (Thomas et al., 2016). To support development of CBL0137 as a possible drug for HAT, we searched for additional lead compounds in the event of unanticipated liabilities of CBL0137. Therefore, we tested the structural analogs CBL0187 (1-[6-acetyl-2,7-dihydroxy-9-[2-(isopropylamino)ethyl] carbazol-3-yl]ethanone) and CBL0174 (1-[6-acetyl-9-[3-(dimethylamino)propyl]-2,7-dihydroxy-carbazol-3-yl]ethanone), which had similar *in vitro* physicochemical, metabolic, and trypanocidal activity. Surprisingly, in a mouse model of chronic HAT, CBL0137 cured the disease but CBL0174 and CBL0187 could not control trypanosome proliferation. While these data expose the failure of current strategies for predicting the efficacy of lead compounds in HAT drug development, they offered an opportunity to reveal new strategies to identify novel leads.

To construct new guidelines to identify carbazole leads for HAT, we made a significant effort to understand differences in the performance of curaxin CBL0137 and its analogs *in vivo*. We found that (i) serum reduces the anti-trypanosome activity of some curaxins, (ii) exposure of curaxins varies in mice, and (iii) the compounds’ biological actions (*i.e.,* molecular pharmacodynamics (PD)) in *T. brucei* are not identical despite their extensive structural similarity (Table 1). We deduced that curaxin’s potential to cure HAT in mice is associated with meeting multiple criteria. The candidate inhibits endocytosis of transferrin and protein synthesis in the trypanosome. The candidate’s exposure (area under the time-concentration curve over 6 h (AUC_0–6h_)) is ten times higher than trypanocidality (DTC_90_), the concentration that is 90% trypanocidal 24-h after a 6-h exposure of trypanosomes to the compound. Five factors, including pharmacokinetics, trypanocidality, tissue distribution, and modes of action in *T. brucei,* were used to develop a multi parameter score for ranking candidate curaxins in lead optimization studies for HAT drug development. A “curaxin HAT lead efficacy (CHLE)” score was calculated using pharmacokinetic, physicochemical, metabolic, brain exposure, and pharmacodynamic data. Principles used to develop CHLE scores may be deployed to calculate new ones for other chemotypes during the discovery of leads for either HAT or different infectious diseases.

## Results

### Physicochemical, catabolic, and anti-trypanosome properties of curaxins

CBL0137 controls parasitemia in mice infected with *T. brucei* (Thomas et al., 2016), so we used its anti-trypanosome and physicochemical properties as the benchmark for selecting lead drugs from this class. CBL0137, CBL0174, and CBL0187 had comparable activity against *T. brucei* Lister 427, with EC_50_ values (the compound concentration that reduced trypanosome proliferation by 50% during a 48-h culture period) of 12.5 ± 2.6, 21 ± 2.8, and 8.9 ± 1.1 nM, respectively (Table 1). Drug-like properties of CBL0137, CBL0174, and CBL0187 (Lipinski et al., 2001; Katsuno et al., 2015; Ferrins et al., 2018) are summarized in Table 1.

CBL0137, CBL0174, and CBL0187 met recommended thresholds for lipophilicity (cLogP ≤ 3; cLogD ≤ 2) (Lipinski et al., 2001; Devine et al., 2017; Ferrins et al., 2018) and chemical properties expected for drug-like molecules (Supplementary Table S2). All compounds exceeded the recommended threshold for aqueous solubility (i.e., greater than 10 μM) with the following values: CBL0137 (992 μM), CBL0174 (773 μM), and CBL0187 (36.3 μM) (Table 1). Metabolic clearance by human liver microsomes (HLM CL_int_) for all three compounds was lower than a recommended threshold of 47 μL/min/mg [CBL0137 (<3 μL/min/mg), CBL0174 (4.89 μL/min/mg), and CBL0187 (12.4 μL/min/mg)].

Intestinal absorption of the three test compounds was predicted using monolayers of human Caco-2 cells (Hubatsch et al., 2007). Apparent permeabilities of CBL0137, CBL0174, and CBL0187 from the apical to the basal side (P_app_ A-to-B) were 19.0 ± 0.42 × 10^−6^ cm/s, 17.8 ± 0.35 × 10^−6^ cm/s, and 14.0 ± 1.91 × 10^−6^ cm/s, respectively. Permeabilities from the basal to the apical side (P_app_ B-to-A) were 22.5 ± 0.14, 21.9 ± 3.39, and 21.7 ± 3.39 × 10^−6^ cm/s, respectively (Table 2). This assay predicts permeability in the intestine and the fraction of the drug absorbed after oral administration, correlating with *in vivo* absorption (Panse and Gerk, 2022). Drugs with P_app_ of less than 1 × 10^−6^ cm/s are categorized as poorly absorbed, those with P_app_ value between 1 and 10 × 10^−6^ cm/s as absorbed moderately, and drugs with P_app_ greater than 10 × 10^−6^ cm/s as well absorbed (Yee, 1997). Thus, all three carbazoles are predicted to possess good *in vivo* absorption characteristics.

In pharmacokinetic studies, plasma levels of all three compounds were determined. Mice received single oral 40, 60, or 50 mg/kg doses of CBL0137, CBL0174, or CBL0187, respectively. These dose levels corresponded to the repeated maximum tolerated dose (rMTD) (see Methods section) of each compound. Plasma samples were obtained at 1, 2, 4, and 6 h post-dosing and analyzed. CBL0137 had the highest plasma concentration of the three compounds: C_max_ of CBL0137, CBL0174, and CBL0187 were 1.86, 1.04, and 0.72 μM in female mice, respectively (Table 3), which were 33-, 28-, and 31-fold higher than EC_50 of_ *T. brucei* AnTat1.1 (Table 1) for corresponding compounds. Male and female mice had similar C_max_ (Supplementary Figure S1) and similar pharmacokinetic profiles (Table 3).

CBL0137, CBL0174, and CBL0187 reached maximum plasma concentrations (C_max_) 3, 1, and 1.5-h post dosing, respectively. T_1/2_ of CBL0137 and CBL0187 could not be determined over 6 h since their concentrations did not drop below 50% of C_max_ over that period. Thus, for CBL0137 and CBL0187, t_1/2_ is greater than 6 h. In the case of CBL0174, t_1/2_ was 3.61 h (Table 3). The mean residence time (MRT_last_) was longest for CBL0137 (3.02 h) and shortest for CBL0174 (2.58 h) (Table 3).

Brain-to-plasma concentration ratios of the carbazoles ranged from 5.3 (CBL0137) to 27.9 (CBL0174) (Supplementary Figure S2). Thus, all curaxins had higher concentrations in the brain than plasma. These data predicted that CBL0137, CBL0174, and CBL0187 would control parasite load in trypanosome infections of mice.

### Trypanocidality of curaxins

**FIGURE 1:**
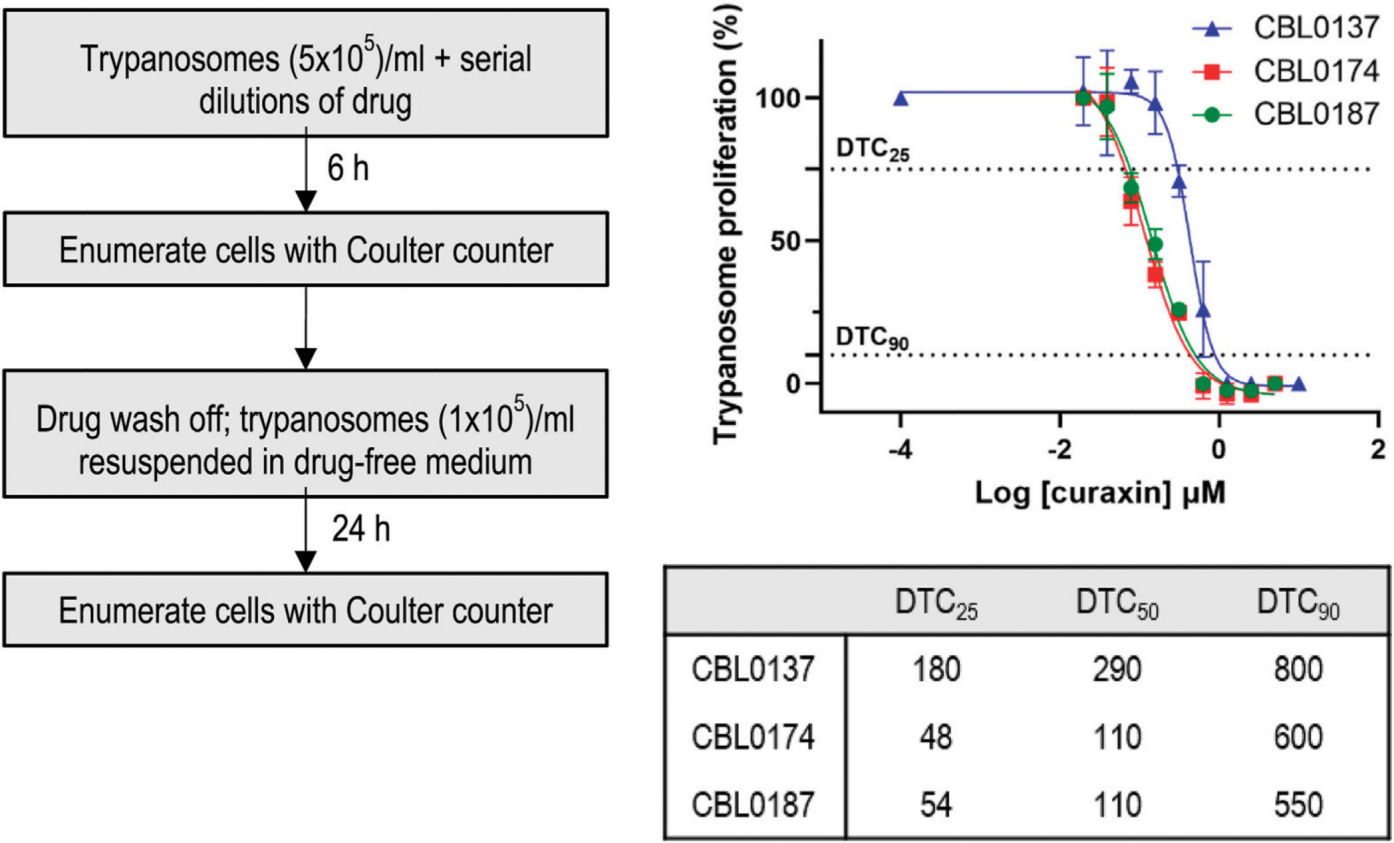
Delayed trypanocidal concentration (DTC) of CBL0137 analogs: *T.b. brucei* (5 × 10^5^/mL HMI-9) was treated with serial dilutions of compounds in 24-well plates for 6 h. Trypanosomes were washed, enumerated with a Coulter Counter, and transferred into a drug-free HMI-9 medium for 24 h in a 24-well plate (1 × 10^5^ cells/mL). After 24 h, cells were enumerated with a Coulter counter (see flow-chart). Data obtained were analyzed using GraphPad Prism to determine DTC_25_, DTC_50_, and DTC_90_ values.

Trypanocidality of the compounds revealed after a short-term cell exposure supports their potential for selection of lead candidates. The premise is that drugs administered to mice are briefly in contact with trypanosomes before elimination. Therefore, we sought hits that, after a 6-h treatment and wash-off, prevented the proliferation of trypanosomes. From dose-response data, compound concentrations that caused 50% cidality (DTC_50_) were: CBL0137, 0.29 μM; CBL0174, 0.11 μM; CBL0187, 0.11 μM (Table 1; Figure 1). Thus, all three compounds appeared to be irreversibly trypanocidal *in vitro.*

#### Justification for using delayed trypanocidal concentrations (DTCs) of curaxins to study their biological effects

Identifying modes of action can be difficult when hits are identified through phenotypic screening (Buckner et al., 2020), and there are no guidelines for systematically addressing this problem, so we devised protocols to assess modes of action for such hits (Sharma et al., 2022). Experiments of this nature require internal controls, so it is important to simplify the comparison of the biological effects of two or more drugs when determining how much of each compound to use per experiment. Most appropriate for such studies are concentrations that were normalized for solubility in culture medium, physicochemical properties, metabolism, and trypanocidality (Bachovchin et al., 2019; Sharma et al., 2022). We therefore determined DTC_25_ concentrations of curaxins (Figure 1; Table 1).

DTC_25_ is the concentration of hit that after 6-h treatment of trypanosomes (and drug wash-off) has no immediate effect on parasite viability but, following a 48-h culture, causes a 25% reduction in number of trypanosomes (Bachovchin et al., 2019; Sharma et al., 2022). To determine DTC_25_, trypanosomes were incubated with various amounts of curaxins for 6 h, rinsed, and then transferred to a drug-free medium for 48 h. The concentration of drug causing a 25% reduction in cell numbers under these conditions (DTC_25_) was determined as 180 nM (CBL0137), 54 nM (CBL0187), and 48 nM (CBL0174) (Figure 1; Table 1). Because DTC_25_ is normalized for the growth of trypanosomes, the amount of hit causing the effect is expected to differ as reported here.

Once the biological effects of hits are determined using DTC amounts, it is very tempting to find the amount of each hit blocking a physiologic effect (*i.e.,* EC_50_). However, for the purpose of determining the importance of a physiologic process in proliferation inhibition, those EC_50_s are not directly relevant to cell division. That is, if at DTC_25_ the event (e.g., endocytosis) is not blocked by the hit, it is difficult to rationalize the event’s relevance for proliferation inhibition by the hit. Thus, those EC_50_ data are best saved for studies focused strictly on the named process (*e.g.,* translation or endocytosis or DNA synthesis).

### Effect of curaxins on endocytosis, DNA replication, and protein synthesis

Close structural analogs can have differing effects on trypanosomes (Begolo et al., 2018). For this reason, we determined whether CBL0137, CBL0174, and CBL0187 prevent the proliferation of trypanosomes via similar mechanisms.

**FIGURE 2:**
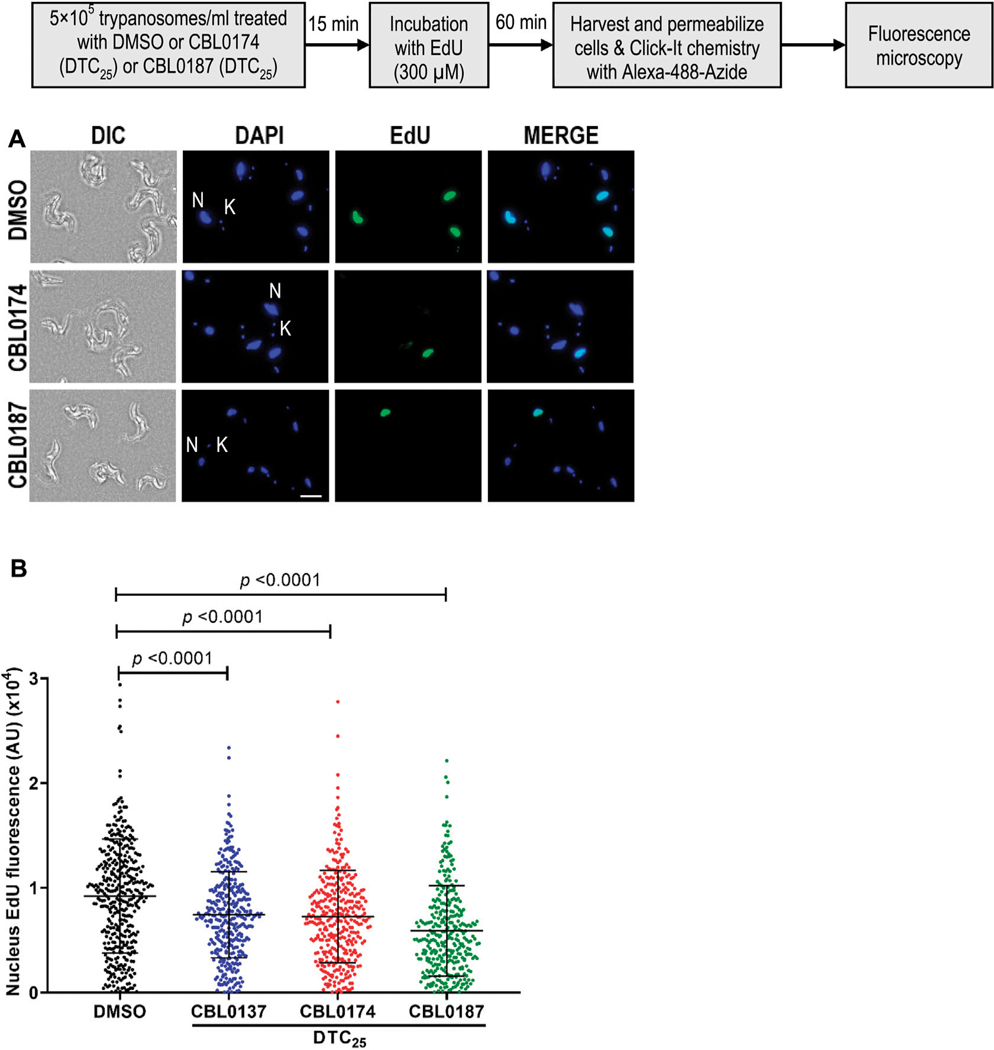
Curaxins reduce DNA synthesis in *Trypanosoma brucei: T. b. brucei* (5 × 10^5^/mL) was treated with DMSO (D: 0.1%), DTC_25_ of CBL0137 (180 nM), CBL0174 (48 nM), or CBL0187 (54 nM) for 15 min and labeled with EdU (300 μM) for 1 h. Incorporated EdU was tracked by clicking with azide-Alexa Fluor-488 and observed with a fluorescence microscope. For quantitation, at least 100 cells were analyzed from each sample. (**A**) Representative images of the EdU-positive signal (green) with DAPI (blue) in 0.1% DMSO (top), CBL0174-treated (middle), or CBL0187-treated cells (bottom). (**B**) Representative images for quantitation of nuclear EdU brightness (nEdU). CellProfiler 3.1.9 was used to extract the EdU fluorescence signal over the DAPI area. Lines represent the median with error bars denoting standard deviation (SD) from three biological replicates. The statistical significance of the distribution of EdU brightness in different treatment groups was analyzed with a Mann–Whitney test.

**FIGURE 3:**
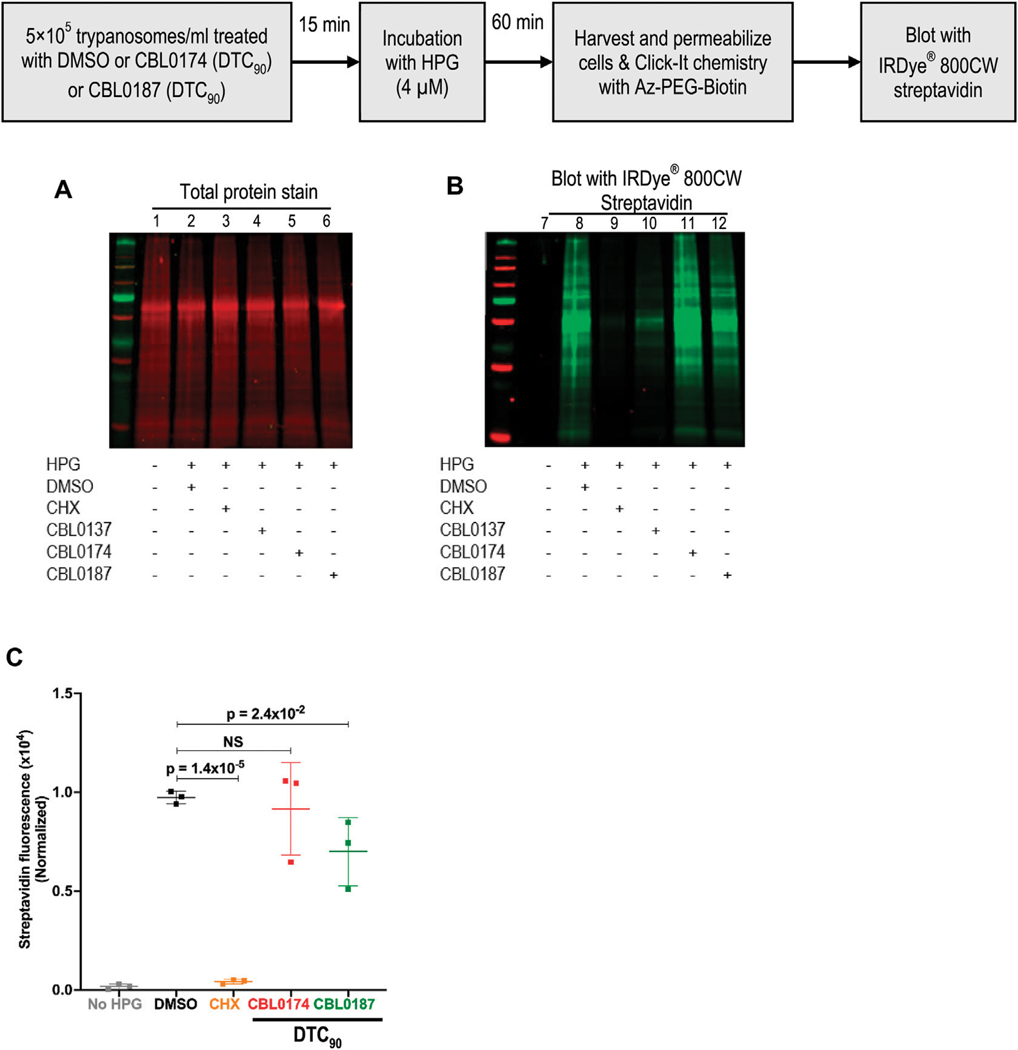
Effect of curaxins on protein synthesis in *Trypanosoma brucei*: *T. b. brucei* (5 × 10^5^/mL) was treated with DMSO (D: 0.1%), DTC_90_ of CBL0174 (550 nM), or CBL0187 (600 nM) for 15 min, followed by 60-min incubation with L-Homopropargylglycine (HPG; 4 μM). Incorporated HPG was clicked to azide-Alexa Fluor-488. Proteins were resolved by SDS-PAGE (12%). **(A)** Proteins were transferred to the PVDF membrane and stained for total protein (using LI-COR^®^ Revert^™^ 700 Total Protein Stain). **(B)** The membrane was blocked with Intercept^®^ (PBS) blocking buffer and reacted with azide-PEG3-biotin conjugate that was detected with Streptavidin IRDye^®^ 800CW (1: 2500 dilution). **(C)** Quantitation of HPG signal normalized to total protein loaded per lane. Statistical significance of differences in mean total HPG incorporation (normalized to total protein) was determined with Student’s t-test.

CBL0137 (DTC_25_) blocks trypanosome DNA replication without affecting protein synthesis (Sanz-Rodriguez et al., 2022). Here, we determined whether CBL0174 or CBL0187 have similar effects on *T. brucei.* New DNA or protein synthesis was tracked by quantitating a thymidine analog 5-ethynyl-2′-deoxyuridine (EdU) incorporation into DNA or of a methionine analog L-homopropargylglycine (HPG) incorporation into protein. Since the molecular effects of CBL0137 have been reported at DTC_25_ (Sanz-Rodriguez et al., 2022), we used equivalent concentrations for biological studies with CBL0174 or CBL0187. CBL0174 (48 nM) and CBL0187 (54 nM) treatment inhibited EdU incorporation into nuclei compared to DMSO-treatment (median EdU fluorescence Wilcoxon’s test *p* < 0.0001) (Figure 2). Neither compound affected protein synthesis at DTC_25_. At higher concentrations (*i.e.,* DTC_90_), CBL0187 inhibited protein synthesis (*p* = 2.4 × 10^−2^) (Figure 3), whereas CBL0174 did not prevent mRNA translation (Figure 3).

**FIGURE 4:**
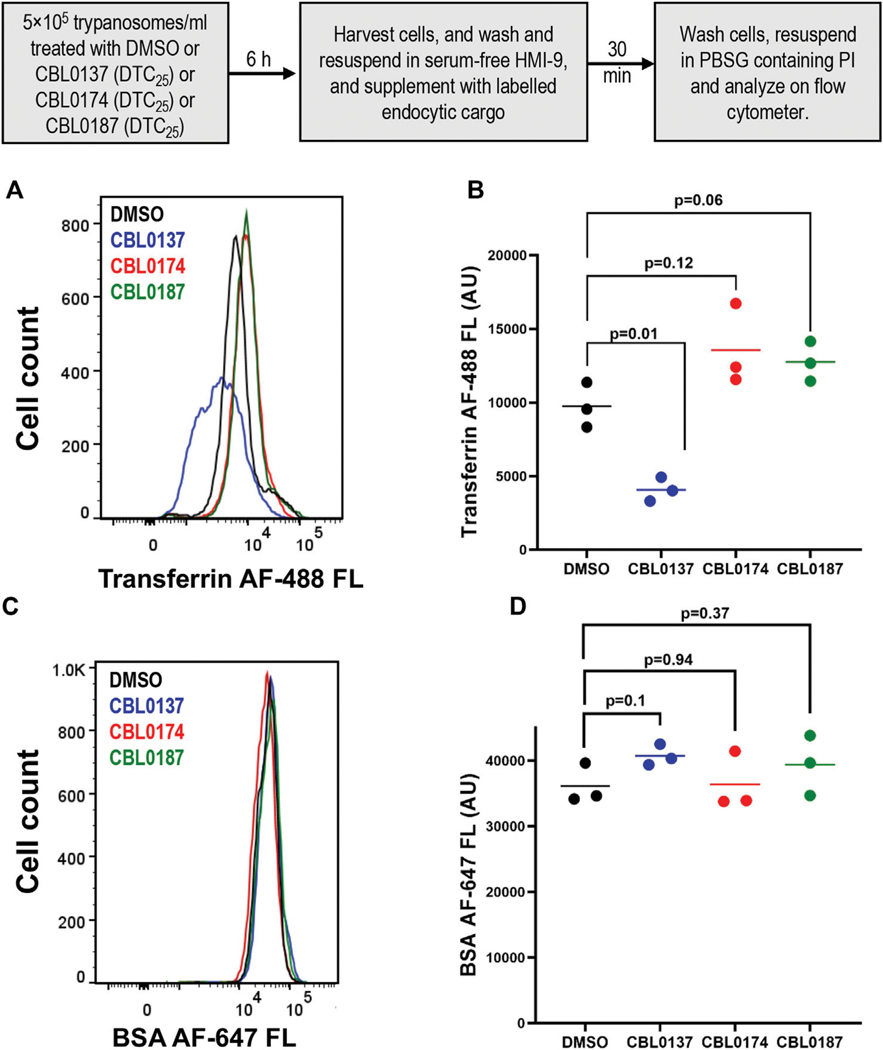
CBL0137, but not CBL0174 and CBL0187, reduces transferrin endocytosis: *Trypanosoma brucei* (5×10^5^ cells/mL) in HMI-9 medium was treated with DMSO (0.1%), or DTC_25_ of each curaxin at for 6 h. Cells were washed and resuspended in serum-free medium. Trypanosomes were incubated with fluorescent endocytosis ligands transferrin (Tf) or bovine serum albumin (BSA) for 30 min (37 °C). A flow cytometer was used to detect fluorescence intensity per cell. Propidium iodide (PI) was used to stain and gate out non-viable cells. Histograms present fluorescence intensity of Tf-AF488 **(A)** or BSA-AF647 **(B)** for every cell (n > 10,000 for each cargo) in DMSO or drug-treated samples. Points on the scatter plot represent mean fluorescence intensities of Tf **(C)** or BSA **(D)** from three biological replicates (calculated with FlowJo). Bars indicate mean ± SD. Student’s t-test was used to evaluate statistical significance of differences in mean intensity of endocytosed ligand in DMSO-treated sample compared to curaxin-treated samples.

We reported in Sanz-Rodriguez et al. (2022) that CBL0137 destabilizes proteins, including pseudokinase NRP1, which is important for the endocytosis of transferrin (Tf) (Kumar et al., 2022). Consequently, we tested the effect of curaxins on the endocytosis of Tf (or a control protein BSA) (Figure 4A). CBL0137 (DTC_25_) reduced the endocytosis of transferrin (Tf) (by 58%) compared to DMSO treatment of trypanosomes (*p* = 0.01, *t*-test) (Figure 4B). In contrast, Tf endocytosis increased 39% after treatment with CBL0174 (*p* = 0.12, *t*-test) or CBL0187 (30.9% increase; *p* = 0.06, *t*-test) (Figure 4B), although these differences were not statistically significant. None of the carbazoles affected BSA endocytosis (Figures 4C, D; Supplementary Table S3). Thus, CBL0137 (DTC_25_) is unique among these curaxins in selectively inhibiting endocytosis of Tf, an essential growth factor for *T.* brucei (Fast et al., 1999).

### CBL0174 and CBL0187 cannot limit proliferation of trypanosomes in mice

**FIGURE 5:**
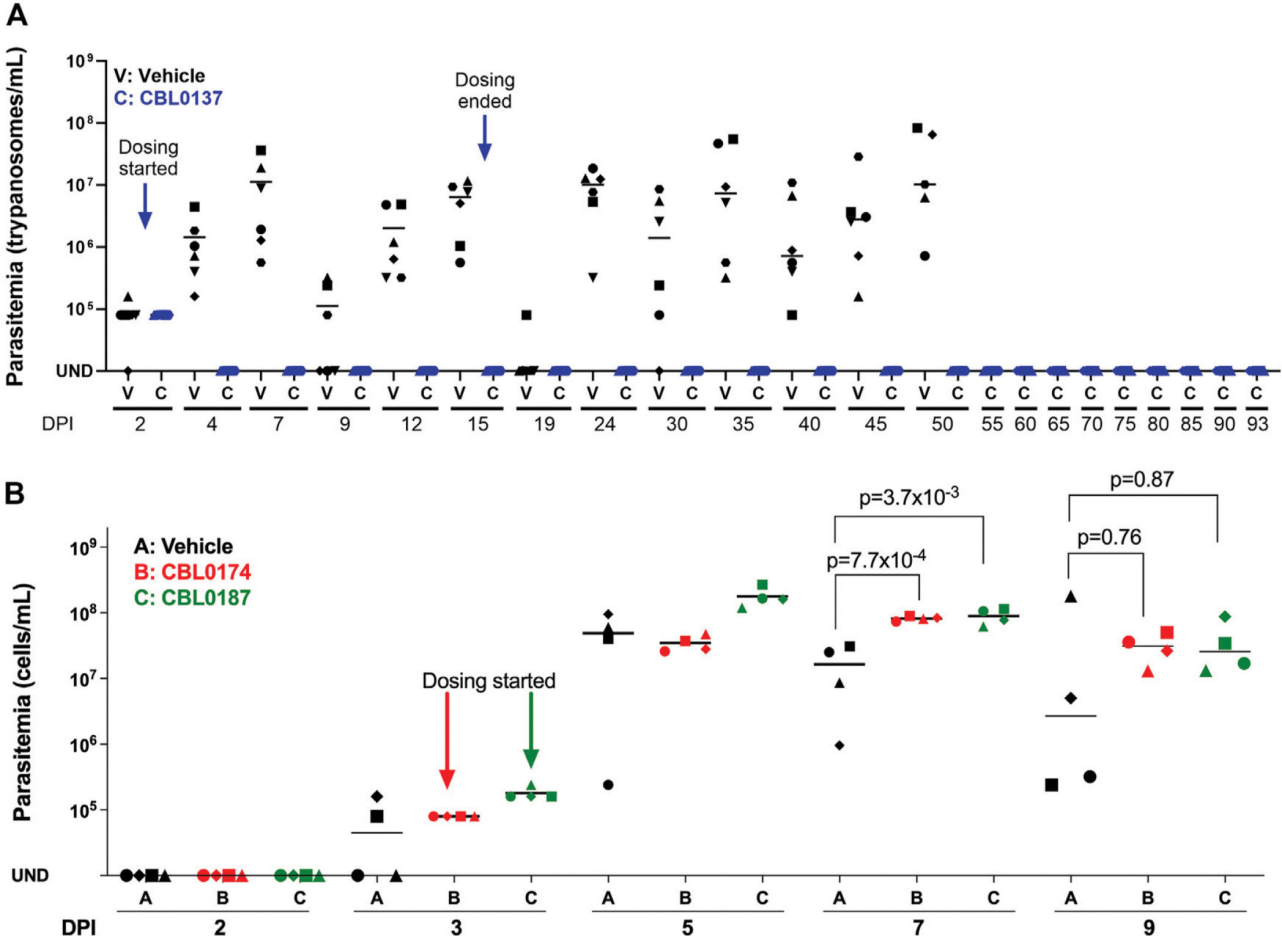
CBL0137 but not CBL0174 or CBL0187 cures chronic HAT in a mouse model of disease. Mice were each infected intraperitoneally with *Trypanosoma brucei* AnTat1.1 (10^4^). **(A)** CBL0137: Group V received the vehicle orally. Group C received CBL0137 (40 mg/kg orally) on days 2, 3, 4, 5, 8, 9, 10, 11, 14, and 15 post-infection. **(B)** CBL0174 and CBL0187: Groups V, B, and D were given vehicle, CBL0174 (60 mg/kg) and CBL0187 (50 mg/kg), respectively, from day 3 through 9. Different shapes represent individual mice, and horizontal lines in each group denote mean parasitemia determined with a hemocytometer. UND = undetectable; DPI = days post-infection.

Since CBL0137 is a lead for anti-HAT drug discovery (Thomas et al., 2016), we tested whether CBL0174 and CBL0187 could serve as backup leads using a chronic HAT model established in mice with a pleomorphic AnTat1.1 strain of *T. brucei* (Claes et al., 2009). At least three waves of parasitemia were observed in mice after infection with *T. brucei* AnTat1.1 (*i.e.,* vehicle-treated), as expected from the phenomenon of antigenic variation (Bangs, 2018). Parasitemia typically peaked between 10^7^ and 10^8^ trypanosomes/mL before receding (Figure 5A) and increasing again (Matthews, 2015). Untreated mice died 45–50 days after infection. In CBL0137-treated mice, no parasites were detected in the blood after two doses of drug (Figure 5A). Parasitemia remained undetectable for at least 93 days (Figure 5A), indicating that the mice were cured of chronic HAT.

CBL0187 and CBL0174 failed to control parasitemia (Figure 5B). Indeed, on day 7 post-infection, the mean parasitemia of CBL0187- (8.99 × 10^7^ ± 2.43 × 10^7^; *t*-test, *p* = 3.7 × 10^−3^) and CBL0174- (8.22 × 10^7^ ± 6.64 × 10^6^; *t*-test, *p* = 7.7 × 10^−4^) treated mice was higher than vehicle-treated mice (1.64 × 10^7^ ± 1.4 × 10^7^) (Figure 5B). The study was terminated on day 9 for humane reasons due to the failure of the compounds to curb the proliferation of trypanosomes in mice.

### C_max_ or AUC_0–6h_ amounts of CBL0187 and CBL0174 are not trypanocidal *in vitro*

**FIGURE 6:**
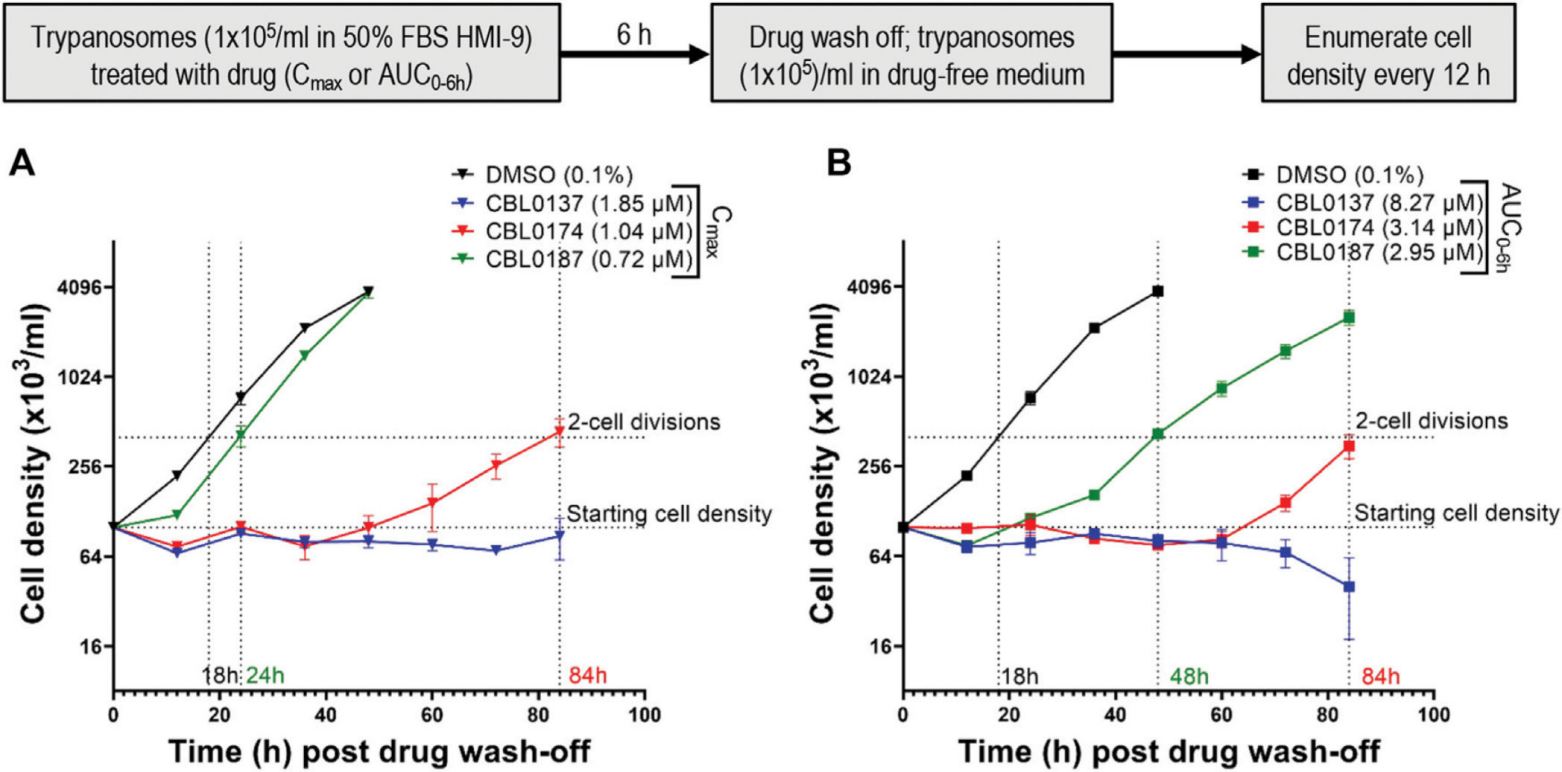
Plasma C_max_ and AUC_0–6h_ of CBL0174 and CBL0187 in mice are not trypanocidal *in vitro*: *Trypanosoma brucei* Lister427 (10^5^ cells/mL) cultured in HMI-9 containing 50% FBS was treated with plasma C_max_ **(A)** or plasma AUC_0–6h_ **(B)** (see Table 5) for 6 h. Trypanosomes were rinsed and resuspended (10^5^ cells/mL) in a drug-free medium, and their density was determined every 12 h for 84 h.

The area under the time curve (AUC) accounts for cumulative drug exposure over 6 hours (AUC_0–6h_) (Table 3). In our studies, AUC is quoted for 6 h for all curaxins, thereby normalizing the time component in the units of measurement and allowing us to focus on the concentration parameter in AUC data. A 6-h time point was selected for our work because it is the doubling time of bloodstream *T. brucei*. AUC_0–6h_s were 8.27 ± 0.58 μM (CBL0137), 3.14 ± 0.25 μM (CBL0174), and 2.95 ± 0.18 μM (CBL0187) (Table 3). Because hits that killed *T. brucei* after only a short exposure seemed the best candidates to advance in lead optimization, *T*. *brucei* was adapted to grow in medium containing 50% serum, after which recovery of cell division after curaxin treatment was monitored. This experimental setup using medium containing 50% serum mimics trypanosome blood better than regular culture medium containing 10% serum (Hirumi and Hirumi, 1994; Creek et al., 2013). We treated trypanosomes for 6 h with curaxin amounts equivalent to either C_max_ or AUC_0–6h_, rinsed the compounds, and determined the lag time for proliferation recovery (set at two cell divisions) (Figure 6).

After treatment with C_max_ equivalents of curaxins (Figure 6A), CBL0137 was trypanocidal because no proliferation of cells was detected after leaving the culture without drug for 84 h (long enough for four cell divisions in 50% serum, as the recovery time of control cells is 18 h). CBL0187-treated trypanosomes resumed proliferation in 24 h (Figure 6A). As the recovery of the proliferation time of control *T. brucei* in 50% serum was 18 h, we subtracted 18 h from the measured doubling time of 24 h and recorded 6 h as the latency period (Table 4). CBL0187 was not trypanocidal, suggesting that it might not perform well in a mouse model of HAT. Likewise, CBL0174 was cytostatic in 50% serum with a delay in the resumption of proliferation of 66 h (Figure 6A; Table 4). These data predict that CBL0174 will not be efficacious in a mouse model of HAT.

The exposure of *T. brucei* to AUC_0–6h_ amounts of curaxins in medium containing 50% serum produced similar results to using C_max_ quantities of curaxins were used. CBL0137-treated trypanosomes failed to proliferate and began dying after 60 h (Figure 6B). The lag in proliferation recovery in culture medium containing 50% serum was 30 h for CBL0187 and 66 h in the case of CBL0174 (Table 4; Figure 6B).

Taken together, these observations (Figure 6) predict data obtained from trypanosome-infected mice treated with curaxins (Figure 5), in that CBL0137 cured the infection, whereas CBL0174 and CBL0187 could not arrest trypanosome proliferation.

## Discussion

### An empirical formula for ranking curaxins as candidates for anti-HAT efficacy studies in a mouse model of disease

African trypanosomes persist in vertebrates because of “antigenic variation” that allows the evasion of immune responses mounted by a host (Bangs, 2018; Pays and Nolan, 2021). Monomorphic *T. brucei* strains (*e.g*., *T. brucei* Lister427) are used routinely in laboratory models of HAT. Infected with these strains, untreated mice die within 10 days, in part because of high parasite loads. In contrast, pleomorphic trypanosomes (*e.g., T. brucei* AnTat1.1) sustain infection for up to 50 days by limiting trypanosome density and by antigenic variation (Faria et al., 2022), with parasitemia occurring in waves (Figure 5A).

Curaxin CBL0137 (40 mg/kg daily oral dose) cured mice infected with pleomorphic *T. brucei* AnTat1.1 (Figure 5A), confirming CBL0137 as a lead for HAT drug development. It is prudent in drug development to have a few backup compounds in case of unforeseen liabilities with the primary lead. Two promising backups for CBL0137 were CBL0174 and CBL0187 (Thomas et al., 2016), both of which have acceptable anti-trypanosome, metabolic, and physicochemical properties (Tables 1, 2; Figure 1). However, neither curaxin controlled parasitemia in mice (Figure 5B).

Using three structure-matched curaxins, we found a combination of factors that were associated with the efficacy of CBL0137 in a mouse model of HAT. Multiple parameters were considered: trypanocidality (Table 1), exposure of trypanosomes to curaxins in mice (Table 3), tissue distribution of curaxins in mice (Supplementary Figure S2), molecular effects of the carbazoles in trypanosomes (Figures 2–4; Supplementary Table S3), and serum shift of EC_50_ (Supplementary Figure S3). From this information, we constructed an empirical formula, the “curaxin HAT lead efficacy” (CHLE) score (defined in the legend of Table 5 and discussed below). CBL0137 had the highest CHLE score.

The rationale for the CHLE formula (Table 5) is as follows. Factors whose values are desirable in large numbers are numerators (e.g., DTC_90_:AUC_0–6h_, and brain–blood distribution ratio). Denominators were parameters preferred in small numbers (*e.g.,* metabolism and residual translation inhibition). Two variables received additional weighting in acknowledgement of their high value in these studies. First, DTC_90_:AUC_0–6h_ was multiplied by 10 to emphasize its importance in pharmacokinetics (PK)/ pharmacodynamics (PD)/ modes of action models. AUC_0–6h_ is obtained from PK work in mice. That value must significantly exceed the trypanocidality (DTC_90_) of the curaxin; otherwise, chances of the hit controlling the proliferation of *T. brucei* in mice is low. Second, we added the brain–blood distribution of curaxins as a denominator because anti-HAT drugs are expected to cross the blood–brain barrier to eliminate parasites in the central nervous system. Tropism of *T. brucei* in vertebrates is undergoing revision (Crilly and Mugnier, 2021), so other tissues harboring trypanosomes may deserve consideration in future iterations of CHLE scores.

We developed a new proliferation recovery assay to complement predictions from CHLE scores. Here, hit concentrations from pharmacokinetics (in mice) are used *in vitro* against cultured trypanosomes. With AUC_0–6h_ amounts of curaxins, CBL0137 was trypanocidal, whereas CBL0187 and CBL0174 delayed cell division by 30 or 66 h, respectively (Figure 6; Table 4). Curaxins with CHLE scores equivalent to or exceeding that of CBL0137 are expected to be lead compounds for HAT drug development. CHLE scores will be used to rank new curaxins (Singh et al., 2023) before efficacy tests in mice for anti-HAT activity.

**FIGURE 7:**
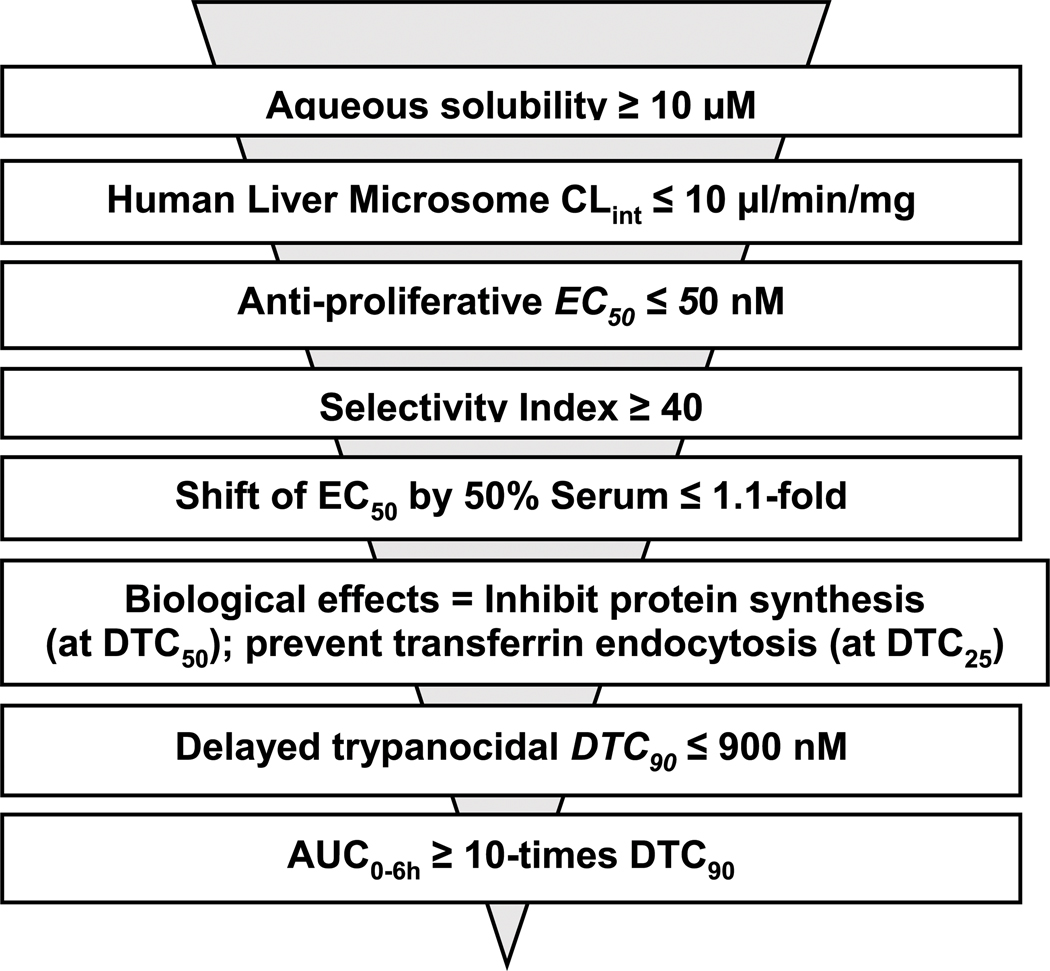
Proposed sequence for determining criteria used for ranking analogs of CBL0137 before testing in mouse model of HAT.

Regarding general applications of our findings, we anticipate that formulae like CHLE scores can be developed by others working with matched hits in other pathogen drug development programs. General guidelines are as follows. CHLE score was calculated with five parameters: (i) ratio of AUC_0–6h_ to DTC_90_, (ii) brain–plasma distribution, (iii) serum shift of EC_50_, (iv) transferrin endocytosis stimulation, and (v) translation inhibition. Parameters (i)–(iii) specify pharmacokinetic and tissue distribution thresholds and may be regarded as constants that all potential leads for the disease must exceed (see traditional screening funnel in Figure 7). In contrast, parameters (iv) and (v) are pharmacodynamic, obtained from pathogen–hit interactions. For that reason, those data are likely to be specific for each chemical class as well as the pathogen under study. The choice of molecular pathways assessed for a chemical scaffold may be determined empirically or can be guided by known functions of the hit’s targets in the pathogen—see Guyett et al. (2016), Huang et al. (2016), Guyett et al. (2017), Sullenberger et al. (2017), Barbosa da Silva et al. (2021), and Meyer and Shapiro (2021) for examples.

The CHLE score has predictive potential: its application calls for data from pharmacokinetics and tissue distribution in addition to pharmacodynamics and metabolism properties. Pharmacokinetic and tissue distribution data are not available for a new set of curaxins (Singh et al., 2023), so the score function cannot yet be applied to that set of compounds.

### Hydroxylation abolishes CBL0137 inhibition of Tf endocytosis

Endocytosis of transferrin in *T. brucei* is affected by multiple factors whose contribution to the process is under investigation (Pal et al., 2003; Subramanya et al., 2009; Guyett et al., 2016; Umaer et al., 2018; Garrison et al., 2021; Reyes-Lopez et al., 2015). Several small-molecule inhibitors of Tf endocytosis are known (Guyett et al., 2016; Sharma et al., 2022).

Curaxins present a fascinating case of how changes on a carbazole scaffold affect biological functions. CBL0137 (DTC_25_) blocked endocytosis whereas CBL0187 (DTC_25_) had no effect on the pathway (Figure 4). CBL0187 (1-[6-acetyl-2,7-dihydroxy-9-[2-(isopropylamino)ethyl]carbazol-3-yl]ethanone) differs from CBL0137 (1-[6-acetyl-9-[2-(isopropylamino)ethyl]carbazol-3-yl] ethanone) in having hydroxyls on the 2 and 7 positions of the carbazole (Table 1) (Thomas et al., 2016). Since the two curaxins are matched structurally, we can directly infer that the hydroxylation of CBL0137 eliminates its inhibition of the endocytosis of Tf. CBL0174 ((1-[6-acetyl-9-[3-(dimethylamino)propyl]-2,7-dihydroxy-carbazol-3-yl]ethanone) is a structural analog of CBL0187 in which the isopropylamine N-substituent on the carbazole is replaced with a dimethylaminopropyl group (Figure 1). CBL0174 (DTC_25_) did not promote endocytosis of Tf (Figure 4). Thus, the effect of hydroxylation of the carbazole scaffold persists after the isopropylamine (in CBL0187) is replaced with a dimethylaminopropyl (in CBL0174).

New studies (*e.g.,* discovery of physiologic targets of curaxins—Mensa-Wilmot (2021); Sanz-Rodriguez et al. (2022)) are needed to provide molecular explanations for the different effects of CBL0137 and CBL0187 on Tf endocytosis.

### Limitations of the study

No agreement exists on the precise features of small molecules to guide their selection to progress in drug development programs. Different chemotypes or pharmacophores may have unique effects on the pharmacodynamic and pharmacokinetic properties of hits. Consequently, conclusions from our studies with curaxins apply to that set of carbazoles used in anti-trypanosome drug development. Whether principles from our work may be applied to other pharmacophores in infectious disease drug discovery will require a new set of studies beyond the scope of this work.

We acknowledge that we were very optimistic in exposing trypanosomes to AUC_0–6h_ or C_max_ in our *in vitro* model of exposure of trypanosomes to curaxins in mice because neither concentration is sustained for 6 h in a mouse infection. Nevertheless, the results from that set of studies is consistent with data from treating infected mice with the three curaxins, giving credence to our newly developed proliferation recovery assay using AUC_0–6h_ or C_max_ amounts of hits to rank candidates for lead compound selection.

## Methods

### Formulation of compounds

The test compounds (CBL0137: 1-[6- acetyl-9-(2-[(propan-2-yl) amino] ethyl)-9H- carbazol-3- yl] ethan-1- one, CBL0174: 1-(6-acetyl-9-[3- (dimethylamino) propyl]-2,7- dihydroxy-9H- carbazol-3-yl) ethan-1- one, and CBL0187: 1-[6- acetyl-2,7- dihydroxy-9-(2-[(propan-2- yl) amino] ethyl)-9H- carbazol-3- yl] ethan-1- one) (Table 1) in the form of hydrochlorides were provided by Incuron, Inc.(Buffalo, NY). Synthesis of the curaxins is described in US patent number PCT/US 2009/059558.

For *in vitro* studies, stock solutions of compounds were prepared in dimethyl sulfoxide (DMSO). The final concentration of DMSO in the culture medium was ≤0.1%. For mouse studies, the compounds were dissolved in a vehicle of *N*-methyl-2-pyrrolidone (10% v/v) and 0.2% hydroxypropyl methylcellulose (90% v/v).

### Physicochemical properties of curaxin analogs

#### Thermodynamic aqueous solubility (pH 7.4).

Various amounts of compounds in powdered form were added to phosphate buffer (0.1 M, pH 7.4). Suspensions were kept on a shaker at 25 °C for 20–24 h. After centrifugation, compounds in the supernatant were quantified using LC-MS/MS.

#### Intrinsic metabolic clearance.

Human liver microsome intrinsic clearance (HLM Cl_int_) was assessed as described (Konsoula and Jung, 2008) at 37 °C for 15 min. The test compound was added to a final concentration of 1 μM, and the residual concentrations were tracked with LC-MS/MS at defined time points.

#### Caco-2 bidirectional permeability assay.

Caco-2 bidirectional permeability assay was performed at Absorption Systems (Exton, Pennsylvania). Briefly, monolayers of Caco-2 cells (clone C2BBe1) were grown to confluence on collagen-coated, microporous membranes in 12-well plates. Permeability assay buffer (Hanks’ balanced salt solution containing 10 mM HEPES and 15 mM glucose, pH 7.4) in the receiver chamber contained 1% bovine serum albumin. Compounds dissolved in assay buffer (5 μM) were added either on the apical (A) or the basolateral side (B) and incubated at 37 °C with 5% CO_2_ in a humidified incubator. Samples were collected from donor and receiver chambers at 120 min. Each determination was performed in duplicate. The flux of lucifer yellow dye was measured post-experimentally for each monolayer to ensure that no monolayers were damaged during the flux period with compounds. Drug amounts were determined by LC-MS/MS. Apparent permeability (P_app_) and percent recovery were determined as per Hubatsch et al. (2007). The efflux ratio was calculated as P_app_ (B-to-A)/P_app_ (A-to-B).

#### Maximum tolerated dose of compounds in mice.

To determine a single maximum tolerated dose (sMTD), male and female Swiss Webster mice were first given a single 50 mg/kg dose of compound formulated in N-methyl-2-pyrrolidone (10% v/v) and 0.2% hydroxypropyl methylcellulose (90% v/v). Mice were monitored daily for up to 7 days post-administration for overall condition using a Total Condition Scoring (TCS) system (Supplementary Table S1) and body weight changes. If greater than 20% body weight loss was recorded, the dose was lowered in two-fold amounts in subsequent tests. If no body weight changes occurred, the single dose was increased to 100 mg/kg, and the mice were observed for up to 7 days. When no toxicity was observed, sMTD values were utilized to determine the repetitive maximum tolerated dose (rMTD). Male and female Swiss-Webster mice were treated with sMTD for 10 consecutive days. If they showed no overt signs of toxicity or greater than 20% body weight loss, the dose was considered the rMTD. If adverse effects were recorded, the daily doses were reduced two-fold in follow-up work. At the end of the study, mice were euthanized by CO_2_ asphyxiation followed by cervical dislocation.

#### Pharmacokinetics.

The plasma concentration and tissue distribution of drugs were determined by Absorption Systems. Briefly, male and female Swiss Webster mice were administered a single 40 mg/kg, 60 mg/kg, or 50 mg/kg dose of CBL0137, CBL0174, or CBL0187, respectively. These dose values corresponded to rMTD for each of these compounds. Blood and brain samples were obtained at 1, 2, 4, and 6 h post-dosing, and plasma and brain concentrations of the drug were determined using LC-MS/MS. Stock standards for calibration were prepared in a matrix comprising equal volumes of water and mouse blood containing K_2_-EDTA as an anticoagulant. Working solutions were prepared in 50:50 acetonitrile–water and added to the matrix to final concentrations of 1,000, 500, 100, 50.0, 10.0, 5.00, 1.00, and 0.500 ng/mL for all compounds. Pharmacokinetic parameters were determined using Phoenix WinNonlin (v8.0) software with sparse sampling. Maximum plasma concentration (C_max_) and the time to reach maximum plasma concentration (t_max_) were determined from the four data points. The area under the time concentration curve (AUC_last_) was calculated using the linear trapezoidal rule with calculation to a 6-h data point. Plasma half-life (t_1/2_) was calculated from 0.693/slope of the terminal elimination phase. Samples below the limit of detection (0.500 ng/mL) were treated as 0.

### Trypanosome culture

*T. brucei* Lister 427 (monomorphic) and *T. brucei* AnTat1.1 (pleomorphic) strains were used here. Pleomorphic *T. brucei* AnTat1.1 used in the study was either adapted for *in vitro* culturing or propagated in mice B6.129S7-Ifng^tm1Ts^/J to obtain blood stabilates. Trypanosomes were maintained in HMI-9 medium (Hirumi and Hirumi, 1989) containing 10% fetal bovine serum (Atlanta Biologicals, Flowery Branch, GA) and 10% Serum Plus (SAFC Biosciences, Lenexa, KS) at densities below 5 × 10^5^ and 1× 10^6^ cells/mL for *T. b.* AnTat1.1 and *T. b.* Lister 427 respectively.

### Trypanosome proliferation inhibition assays

*T. b.* Lister 427 or *T. b.* AnTat1.1 were seeded at 4 × 10^3^ cells/mL or 2 × 10^3^ cells/mL (in 50 μL culture medium), respectively, into 384-well black plate (Greiner; Frickenhausen, Germany) and treated with serial dilutions of each compound in duplicate. Plates were incubated for 48 h at 37 °C and 5% CO_2_. Cells were lysed by adding 15 μL of 1× SYBR Green I (Invitrogen; Carlsbad, CA) containing lysis solution (30 mM Tris pH 7.5, 7.5 mM EDTA, 0.012% saponin, and 0.12% Triton X-100) to each well, followed by incubation in the dark for 1 h at room temperature (Faria et al., 2015). Fluorescence in lysates was quantitated with a fluorometer (Fluoroskan Ascent Microplate Fluorometer, Thermo Scientific) (100 ms; ex: 485 nm, em: 538 nm). Each experiment was performed in triplicate. Non-linear regression plots of data were constructed with GraphPad Prism (GraphPad Software; La Jolla, CA) and used to determine EC_50_ (drug concentration that inhibits trypanosome proliferation by 50%).

### Delayed trypanocidal assay

Mid-logarithmic growth-phase *T. b.* Lister427 were inoculated into HMI-9 medium (5 × 10^5^ cell/mL). Cells were incubated with either 0.1% DMSO (vehicle) or serial dilutions of drug in 24-well plates for 6 h at 37 °C/5% CO_2_, following which trypanosomes were pelleted (3,000 g, 5 min), rinsed with drug-free HMI-9 medium, and cells enumerated with a Coulter counter. Trypanosomes were then inoculated at 1 × 10^5^ cells/mL in 24-well-plates or 384-well plates and cultured for 24 h, after which trypanosomes were checked for viability with a hemocytometer and enumerated with a Coulter counter. Each experiment was performed thrice, and non-linear regression graphs plotted with GraphPad Prism were used to obtain DTC_50_ values (Bachovchin et al., 2019).

### DNA synthesis

Bloodstream trypanosomes (5 × 10^5^ cells/mL) pretreated with DTC_25_ concentrations of CBL0174 or CBL0187 in HMI-9 medium (for 15 min) were incubated with 5-ethynyl-2′-deoxyuridine (EdU) (Abcam) (300 μM) for 1 h. Cells were prepared as per Sullenberger et al. (2017). Images of trypanosomes were captured using a DeltaVision II Olympus inverted microscope processed with Fiji (ImageJ, v 2.0.0) and analyzed using CellProfiler 3.1.9 (Sanz-Rodriguez et al., 2022).

### Protein synthesis

Mid-logarithmic phase trypanosomes were rinsed and resuspended (5×10^5^ cells/mL) in methionine-free RPMI supplemented with 10% fetal bovine serum (FBS). Cells were treated with DTC_25_ or DTC_90_ concentrations of CBL0174 or CBL0187 for 15 min, followed by 1-h incubation with 4 μM (final concentration) L-Homopropargylglycine (HPG) (Cayman Chemical). Cells were washed with PBS-G (PBS containing 10 mM glucose), pelleted, and resuspended (~5 × 10^6^ cells) in 50 μL of permeabilization buffer (50 mM HEPES pH 7.4, 0.025% NP-40) on ice for 30 min. The pellet was mixed with 50 μL “Click-iT reaction” cocktail (20 mM Tris buffer, pH 7.4, 5 mM CuSO_4_, 300 mM ascorbic acid, 140 mM NaCl, and 17.5 μM azide-PEG-biotin) at room temperature for 1 h. Proteins were precipitated by adding 400 μL of ice-cold acetone, resuspended in polyacrylamide gel electrophoresis (PAGE) sample buffer (~1×10^5^ cells/μL), and separated by SDS-PAGE (12%). HPG-labeled proteins were detected with Streptavidin IRDye^®^ 800CW (1: 2500) and visualized on a Li-Cor^®^ Odyssey CLx near-infrared fluorescence imaging system (Sanz-Rodriguez et al., 2022).

### Endocytosis of proteins

Curaxin or DMSO-treated trypanosomes (5 × 10^5^/mL) were washed with serum-free HMI-9 medium (500 uL). Trypanosomes were resuspended in serum-free HMI-9 medium (100 μL) supplemented with either transferrin–AlexaFluor647 conjugate (50 μg, Invitrogen, Eugene, OR) or BSA–AlexaFluor647 conjugate (50 μg, Invitrogen, Eugene, OR) for 30 min at 37 °C, 5% CO_2_. Cells were transferred to an ice-water bath, washed with cold PBS/G at 4 °C (3,000 g for 5 min), resuspended in PBS/G (500 μL) containing propidium iodide (3 μM), and analyzed on a flow cytometer (Beckman Coulter CytoFLEX S). FlowJo software (FlowJo, LLC) was used to gate trypanosomes based on size and shape (forward and side scatter features), and fluorescence intensity of endocytosed protein was measured in viable cells (negative for propidium iodide uptake). FlowJo software was used to determine the mean fluorescence intensity of each cargo in trypanosomes (at least 10,000 events). Data from three independent biological experiments were analyzed with a two-tailed Student’s t-test with unequal variance to determine the possible statistical significance of differences in means of fluorescence intensities of endocytosed cargo.

### Treatment of chronic HAT in a mouse model of disease

Drug efficacy studies in a mouse model of HAT were conducted following recommendations in the “Guide for the Care and Use of Laboratory Animals” of the National Institutes of Health. The protocol was approved by the Institutional Animal Care and Use Committee (IACUC) at the University of Georgia. After the study, the mice were euthanized by CO_2_ asphyxiation followed by incision to form a bilateral pneumothorax, as approved by the American Veterinary Association.

Female Swiss-Webster mice (20–25 g; 8–10 weeks old) were acclimatized for 1 week before the commencement of the study. For the CBL0137 efficacy study, mice (n = 12) were infected intraperitoneally with 1 × 10^4^ *T. brucei* AnTat1.1 and randomly distributed into two groups on day 0. After microscopic confirmation of infection on day 2, the treatment group received a single oral dose of CBL0137 (40 mg/kg) on days 2, 3, 4, 5, 8, 9, 10, 11, 14, and 15. For work with CBL0174 and CBL0187, mice (n = 12) were infected intraperitoneally with 1×10^4^ pleomorphic *T. brucei* AnTat1.1 and randomly distributed into three groups on day 0. After confirming parasitemia by hemocytometry on day 3, mice in treatment groups B and C were given either one oral dose of CBL0174 (60 mg/kg) or CBL0187 (50 mg/kg), respectively, on days 3, 4, 5, 6, 7, 8, and 9. In both studies, untreated mice were infected with 1 × 10^4^ trypanosomes and then received vehicle alone. Parasites in blood collected from a tail venipuncture and diluted 8-fold in RBC lysis buffer (Qiagen) were monitored with a hemocytometer (Neubauer Bright-line). During the study, mice with parasitemia above 10^8^ trypanosomes/mL were euthanized for humane reasons. Mice without parasitemia for 90 days were considered cured.

### Effect of 50% serum on curaxin inhibition of trypanosome proliferation

HMI-9 medium (Hirumi and Hirumi, 1989) was prepared with different final concentrations of fetal bovine serum (FBS) (20, 35, and 50%). *T. brucei* Lister 427 was cultured in each medium for at least 7 days. Thereafter, EC_50_ of CBL0137, CBL0174, or CBL0187 was determined in the modified media as described earlier (Supplementary Figure S3). EC_50_ measurements were obtained from three independent experiments in each medium. Possible statistical significance of differences in EC_50_ values for control (20% FBS containing) and modified (35 or 50% FBS containing) media was determined using Student’s t-test.

### Recovery of trypanosome proliferation after a 6-h exposure to curaxins

The *in vivo* plasma exposure of trypanosomes to drug is expressed as area under the curve (AUC) in pharmacokinetic analysis. Theoretically, the same drug exposures *in vitro* and *in vivo* will be achieved within 6 h if the *in vitro* compound concentration × 6 h value was equal to *in vivo* AUC_0–6h_. We hypothesized that for a compound to be effective *in vivo*, a 6-h *in vitro* exposure of *T. brucei* to a concentration equaling AUC_0–6h_ corrected for plasma protein binding (AUC_0–6h unbound_) ought to halt proliferation of the cells. AUC_0–6h unbound_ is equal to the AUC_0–6h_ × fraction of drug unbound to plasma (PF_U_) (Table 4).

Trypanosomes (1 × 10^5^/mL) were treated with a concentration equivalent of the AUC_0–6h unbound_ (Table 4) of each compound. After 6 h, cells were washed and resuspended (1 × 10^5^ cells/mL) in drug-free medium. Trypanosome densities were determined every 12 h thereafter using a hemocytometer. Since the density of control trypanosomes (treated with 0.1% DMSO) increases four-fold in 12 h, we considered any inhibition of proliferation “recovered” when trypanosome density reached 4× 10^5^/mL. In a complementary study, trypanosomes cultured in media containing 10%, 20%, 35%, or 50% FSB and then treated with C_max_ or AUC_0–6h_ (Table 3) for 6 h. Proliferation recovery time was recorded post drug wash-off as described above.

### Quantification and statistical analysis

Data for all biological assays were obtained from three independent experiments with technical duplicates. Statistical tools and *p*-value thresholds for experiments are described in respective sections; data were analyzed using GraphPad Prism version 9.0 (GraphPad, San Diego, CA).

## Supplementary Material

Supplementary Material

## Figures and Tables

**TABLE 1:** Physicochemical, metabolic, and anti-trypanosome properties of curaxins.

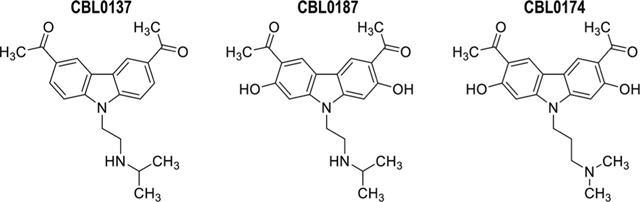			
Property	CBL0137	CBL0187	CBL0174
Aqueous solubility (μM)	992	36.3	773
HLM Cl_int_ (μL/min/mg)	<3	12.4	4.89
EC_50_ *T. brucei Lister427* (nM)	12.5	8.9	21
DTC_25_ *T. brucei Lister427* (nM)	180 (165)	54 (41)	48 (60)
DTC_50_ *T. brucei Lister427* (nM)	290 (278)	110 (98)	110 (122)
DTC_90_ *T. brucei Lister427* (nM)	800 (800)	550 (592)	600 (513)

EC_50_ is the concentration of curaxin that inhibits proliferation by 50% when trypanosomes (4 × 10^3^/mL) are exposed to the compound continuously for 48 h.

DTC is the delayed trypanocidal concentration of test compounds. Here, curaxin treatment is performed for 6 h with trypanosomes (5 × 10^5^/mL). After drug rinse-off, a trypanosome culture is initiated at a density of 1 × 10^5^ cells/mL and survival is tracked for 24 h. DTC_50_ inhibits trypanosome proliferation by 50% after drug wash off.

* Values in parenthesis are delayed trypanocidal concentration obtained from assay where the starting cell density of trypanosomes was 1 × 10^5^/mL instead of 5 × 10^5^/mL as mentioned above.

**TABLE 2:** Cell permeability properties of CBL0137 analogs.

Compound	Direction	Recovery (%)	P_app_ (10^−6^ cm/s)
CBL0137	A-to-B	64	19.0 ± 0.42
	B-to-A	85	22.5 ± 0.14
CBL0174	A-to-B	60	17.8 ± 0.35
	B-to-A	86	21.9 ± 3.39
CBL0187	A-to-B	47	14.0 ± 1.91
	B-to-A	64	21.7 ± 3.39

Caco-2, bidirectional permeability assay was performed as per *Materials and Methods*. Apparent permeability (Papp) and percent recovery were determined as per Hubatsch et al. (2007). The efflux ratio was calculated as Papp (B-to-A)/Papp (A-to-B).

**TABLE 3:** Pharmacokinetic properties of CBL0137, CBL0174, and CBL0187 in Swiss-Webster mice after a single oral dose of 40 mg/kg, 60 mg/kg, and 50 mg/kg, respectively.

	Male			Female		
	CBL0137	CBL0174	CBL0187	CBL0137	CBL0174	CBL0187
C_max_ (μM)	1.89 ± 0.19	1.06 ± 0.06	0.96 ± 0.05	1.86 ± 0.17	1.04 ± 0.08	0.72 ± 0.11*
t_max_ (h)	2.0 ± 0	1.0 ± 0	2.0 ± 0	3.0 ± 1.4	1.0 ± 0	1.5 ± 0.7
t_1/2_ (h)	>6	4.33	>6	>6	3.61	>6
MRT_0–6h_ (h)	3.22	2.72	2.82	3.01	2.58	2.78
AUC_0–6h_ (h. μM)	8.28 ± 0.54	3.44 ± 0.39*	3.59 ± 0.23*	8.27 ± 0.58	3.14 ± 0.25*	2.95 ± 0.18*

C_max_: Maximum plasma concentration.

t_max_: Time of maximum plasma concentration.

t_1/2_: Half-life in plasma.

MRT_0–6h_: Mean residence time, calculated to the 6 h time point.

AUC_0–6h_: Area under the time curve, calculated to the 6 h time point.

ND: not determined due to a lack of quantifiable data points trailing the c_max_.

* Values differ significantly from CBL0137; Student’s t-test *p* < 0.05.

**TABLE 4:** Latency in proliferation recovery (h) of trypanosomes after 6-h treatment with C_max_ or AUC_0–6h_ curaxins in medium containing 50% serum.

	Concentration of curaxin	Latency of proliferation (h)
C_max_	CBL0137 (1.85 μM)	NR
	CBL0174 (1.04 μM)	66 h
	CBL0187 (0.72 μM)	6 h
AUC_0–6h_	CBL0137 (8.27 μM)	NR
	CBL0174 (3.14 μM)	66 h
	CBL0187 (2.95 μM)	30 h

NR, No recovery of trypanosome division 84 h after drug wash-off.

**TABLE 5:** Curaxin HAT lead efficacy (CHLE) scores for CBL0137, CBL0187, and CBL0174.

Curaxin	DTC_90_: AUC_0–6h_	Residual Translation*	Brain to plasma	Shift of EC_50_ in 50% serum	Reduction of Tf uptake**	Score
CBL0–137	10.3	0.05	7.1	1.1	0.58	3,452.3
CBL0–187	5.3	0.72	15.9	4.1	1.31	17.8
CBL0–174	5.4	0.94	26.9	1.5	1.39	41.3

* Determined with DTC_90_ of curaxins.

** Uses DTC_25_ of curaxin.

An empirical formula was used to calculate a multiparameter Curaxin HAT, lead efficacy score = ([(DTC_90_:AUC_0–6h_ ratio) × 10) + (brain–plasma concentration ratio)]/[(relative inhibition of transferrin uptake by curaxin (DTC_25_)) × (shift of EC_50_ in medium containing 50% serum) × residual translation after curaxin (DTC_50_) treatment]. See Discussion for rationale of the formula. DTC_90_: DTC is delayed trypanocidal. DTC_90_ inhibits trypanosome proliferation by 90% after drug wash-off. Trypanosomes (5 × 105/mL) are treated for 6 h with curaxin. After drug rinse-off, a trypanosome culture is initiated at density of 1 × 10^5^ cells/mL and survival is tracked for 24 h.

EC_50_: Concentration of curaxin that inhibits proliferation by 50% when trypanosomes (4 × 10^3^/mL) are incubated with the compound for 48 h at 37 °C. Shift of EC_50_ in 50% serum: ratio of EC_50_ in 20% serum to EC_50_ in 50% serum (see Supplementary Figure S3).

AUC_0–6h_: Area under the time curve (h.μM) calculated to the 6 h time point in pharmacokinetic studies (see Table 3).

Residual Translation: documents effect of curaxins on protein synthesis. *T. b. brucei* (5 × 10^5^/mL) was treated with DMSO (D: 0.1%) or DTC_90_ of curaxin for 15 min, followed by 60-min incubation with L-Homopropargylglycine (HPG; 4 μM) (see Figure 3C and legend for Figure 3). The percentage of curaxin inhibition of translation, compared to control DMSO-treated, is subtracted from 1.

Brain: plasma: ratio of brain concentration of curaxin (ng/g) compared to plasma concentration (see Supplementary Figure S2).

Tf uptake: effect of curaxins (DTC_25_) on endocytosis on transferrin is determined in comparison to a DMSO control (see Figure 4). Stimulation of endocytosis is recorded; inhibition receives a value less than 1. When transferrin uptake is stimulated by the curaxin, fractional increase has a value higher than 1.

## Data Availability

The original contributions presented in the study are included in the article/Supplementary Material; further inquiries can be directed to the corresponding author.
