# Supplementary material for: Multiparameter ranking of carbazoles for anti-trypanosome lead discovery
Source: Front Drug Discov (Lausanne). Author manuscript; Available in PMC 2025 Mar 21. (PMC11927960; doi:10.3389/fddsv.2024.1430927)
Supplement: Supplementary Material [file NIHMS2057386-supplement-Supplementary_Material.pdf]

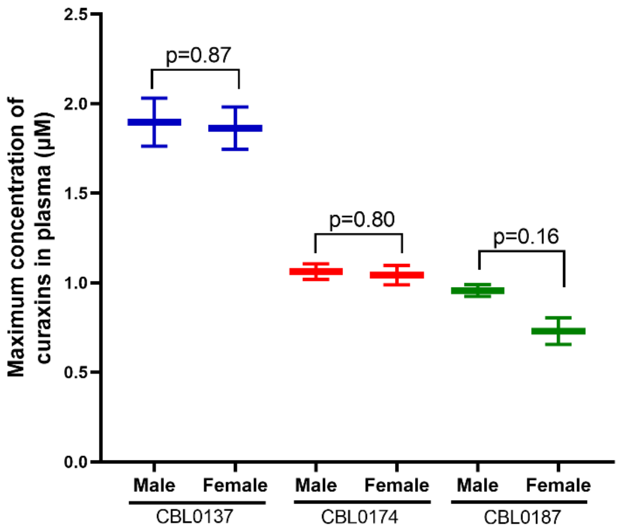

Figure S1: Comparison of plasma C_{max} of curaxins in male and female Swiss-webster mice.

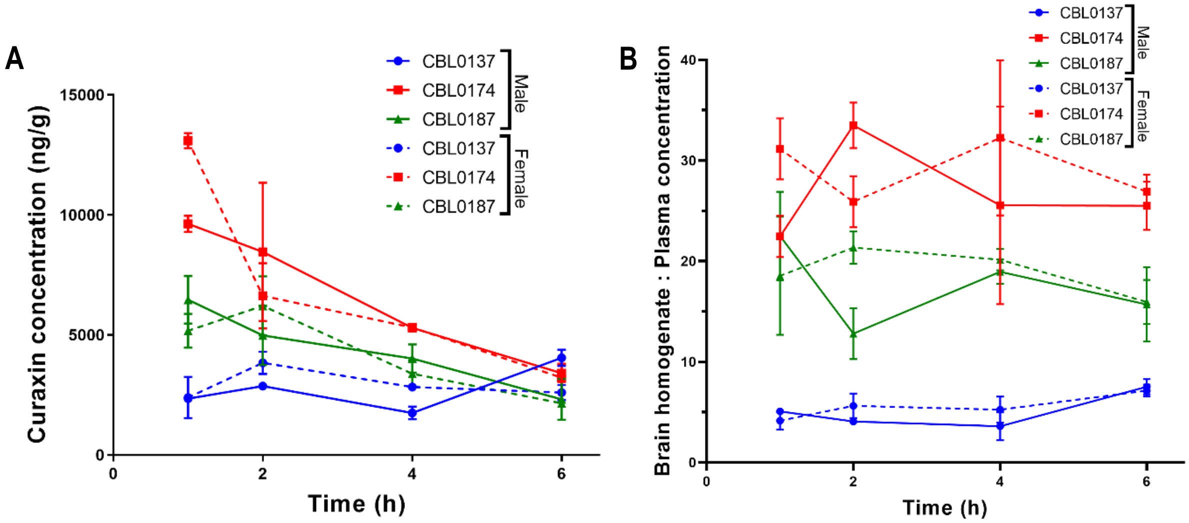

Figure S2: Brain accumulation of Curaxins in mice: Male and female Swiss Webster mice were administered a single 40 mg/kg, 60 mg/kg, or 50 mg/kg dose of CBL0137, CBL0174, or CBL0187, respectively. Blood and brain samples were obtained at 1, 2, 4, and 6 h post-dosing, and plasma and brain concentrations of the carbazoles were determined using LC-MS/MS. **(A)** Brain tissue concentrations of curaxins at different time points **(B)** Ratios of concentrations of CBL0137, CBL0174 and CBL0187 in brain homogenate and plasma of male and female Swiss-wester mice.

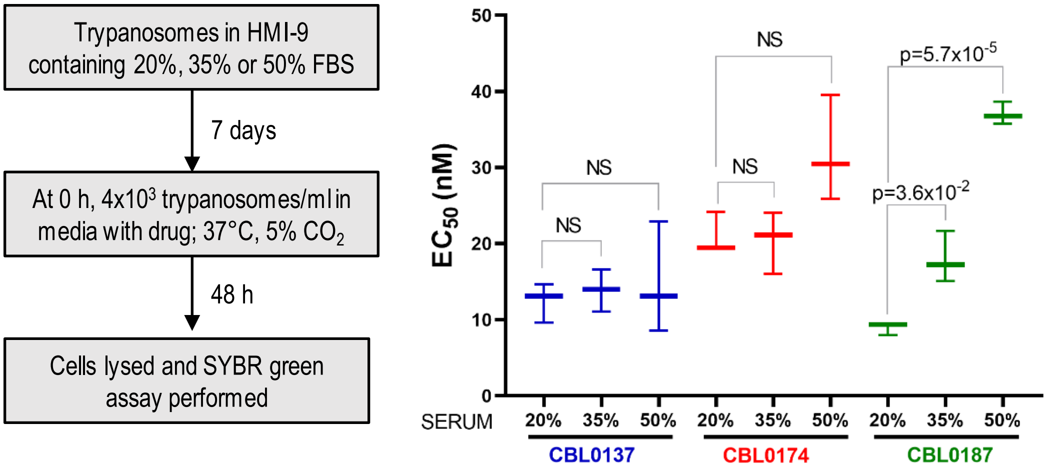

Figure S3: Serum decreases anti-trypanosome potency of CBL0187. HMI-9 (Hirumi and Hirumi, 1989) was modified to contain 20, 35, or 50% fetal bovine serum (FBS) (total concentration). *T. b. brucei* Lister 427 was first cultured in the medium for 7 days. Trypanosomes (4×10^3/mL) were incubated with serial dilutions of analog in different media (50 µL). Plates were incubated for 48 h at 37 °C and 5% CO_2, and the proliferation of trypanosomes was measured using a SYBR green assay (Faria et al., 2015). Each experiment was performed thrice, and non-linear regression fits to the data were obtained using GraphPad Prism which was used to calculate EC_{50} values. The possible statistical significance of the difference in EC_{50} values obtained from assays with 35% or 50% FBS-medium from 20% FBS-medium (control) was determined by Student's T-test.

Table S1: Semi-quantitative total condition scoring system for mice used in the study

Parameter	Score	Observation	Comments
Body Condition	4	Normal	-
	3	Slightly Abnormal	Slightly loose skin, decreased fat or slight abdominal distention
	2	Abnormal	Loose skin & thin, palpable iliac crests & vertebrae, moderate
	1	Severely Abnormal	No palpable fat, severely reduced muscle mass, severe abdominal distention
Posture	4	Normal	-
	3	Slightly Hunched	-
	2	Moderately Hunched	-
	1	Severely Hunched	-
Activity	4	Normal	-
	3	Slightly Lethargic	Less interested in surroundings than normal, but still responsive
	2	Moderately Lethargic	Not interested in surroundings, but still responsive
	1	Severely Lethargic	Not interested in surroundings, not responsive

Table S2: Chemical properties of CBL0137, CBL0174, or CBL0187

	**CBL0137**	**CBL0174**	**CBL0187**
Formula	$C_{21}H_{24}N_2O_2$	$C_{21}H_{24}N_2O_4$	$C_{21}H_{24}N_2O_4$
Composition	C (74.97%),<br>H (7.19%),<br>N (8.33%),<br>O (9.51%)	C (68.46%),<br>H (6.57%),<br>N (7.60%),<br>O (17.37%)	C (68.46%),<br>H (6.57%),<br>N (7.60%),<br>O (17.37%)
Molecular weight (g/mol)	336.435	368.433	368.433
logP	0.4	-0.1	0.2
H-bond donors	1	2	3
H-bond acceptors	4	6	6
Lipinski Rule of 5	4 of 4	4 of 4	4 of 4
logD	0.4	-0.1	0.2
logS	-2.2	-2.1	-2.3
pK_a	11.1 (Basic)	8.7 (Acidic)<br>11.0 (Basic)	8.7 (Acidic)<br>11.1 (Basic)
CNS MPO score	4.8	4.4	4.1
Topological polar surface area (TPSA)	51.1 Å²	82.8 Å²	91.6 Å²
Fsp3	0.33	0.33	0.33
Heavy atom count	25	27	27
Rotatable bonds	6	6	6

LogP (Partition Coefficient) an indicator of a compound's hydrophobic (lipophilic) or hydrophilic nature. A higher logP value suggests higher lipid solubility.
LogD (Distribution Coefficient) is used to measure the lipophilicity of ionizable compounds.
LogS (Aqueous Solubility) is compound's aqueous solubility.
CNS MPO (central nervous system multiparameter optimization) a scoring matrix determined based on ClogP, ClogD, Molecuar weight, TPSA, HBD and pK_a
Fsp3 (Fraction of sp3 Hybridized Carbon Atoms) is a measure of the proportion of carbon atoms in a molecule that are sp3 hybridized (tetrahedral geometry) as opposed to sp2 hybridized (planar geometry). A higher Fsp3 value is generally associated with compounds that resemble natural products and have better drug-like properties.

Table S3: Summary of biological pathways affected by curaxins

	DNA Synthesis (DTC_{25})	Endocytosis (DTC_{25})		Protein Synthesis (DTC_{50})
		Transferrin	BSA	
**CBL0137**	Yes	Inhibited	No	Yes
**CBL0174**	Yes	Activated	No	No
**CBL0187**	Yes	Activated	No	No
